# Catch trials in force field learning influence adaptation and consolidation of human motor memory

**DOI:** 10.3389/fnhum.2014.00231

**Published:** 2014-04-21

**Authors:** Christian Stockinger, Anne Focke, Thorsten Stein

**Affiliations:** YIG “Computational Motor Control and Learning”, BioMotion Center, Institute of Sports and Sports Science, Karlsruhe Institute of TechnologyKarlsruhe, Germany

**Keywords:** reaching movements, dynamic perturbation, intermittent practice, variability, interference, internal model formation, control strategy, robotic manipulandum

## Abstract

Force field studies are a common tool to investigate motor adaptation and consolidation. Thereby, subjects usually adapt their reaching movements to force field perturbations induced by a robotic device. In this context, so-called catch trials, in which the disturbing forces are randomly turned off, are commonly used to detect after-effects of motor adaptation. However, catch trials also produce sudden large motor errors that might influence the motor adaptation and the consolidation process. Yet, the detailed influence of catch trials is far from clear. Thus, the aim of this study was to investigate the influence of catch trials on motor adaptation and consolidation in force field experiments. Therefore, 105 subjects adapted their reaching movements to robot-generated force fields. The test groups adapted their reaching movements to a force field A followed by learning a second interfering force field B before retest of A (ABA). The control groups were not exposed to force field B (AA). To examine the influence of diverse catch trial ratios, subjects received catch trials during force field adaptation with a probability of either 0, 10, 20, 30, or 40%, depending on the group. First, the results on motor adaptation revealed significant differences between the diverse catch trial ratio groups. With increasing amount of catch trials, the subjects' motor performance decreased and subjects' ability to accurately predict the force field—and therefore internal model formation—was impaired. Second, our results revealed that adapting with catch trials can influence the following consolidation process as indicated by a partial reduction to interference. Here, the optimal catch trial ratio was 30%. However, detection of consolidation seems to be biased by the applied measure of performance.

## Introduction

Motor learning is an important attribute of human life which refers to an improvement in execution of a motor behavior. Thereby, motor learning implies two distinct features: the ability to acquire new motor skills and the adaptation of existing motor skills to new environmental conditions (Huang and Krakauer, 2009; Krakauer and Mazzoni, 2011; Kitago and Krakauer, 2013). In neuroscience, motor learning has most often been studied in the context of adaptation of reaching movements. Thereby, subjects usually adapt their reaching movements to either kinematic perturbations (visuomotor rotations, Krakauer et al., 2005; prism-induced displacements, Held and Freedman, 1963) or dynamic perturbations (robot-induced forces, Shadmehr and Mussa-Ivaldi, 1994; rotation of body, Lackner and DiZio, 2005; attached inertial loads, Krakauer et al., 1999). Here, we want to focus on motor learning in terms of adaptation of reaching movements to robot-induced forces. Thereby, subjects commonly interact with a robotic device that applies perturbing forces to the subjects' hands leading to changed dynamic conditions of the reaching movements. At the beginning of reaching under these changed dynamics, subjects' hand trajectories are deviated from desired straight hand paths showing a hooking pattern. This results in a motor error arising from the discrepancy between prediction and execution of the movement. When further exposed to this perturbation, subjects' performance initially improves rapid followed by a slower increase to steady state close to baseline performance (Shadmehr et al., 2010). This kind of fast trial-by-trial reduction of motor errors following an abrupt change in conditions is typically referred to as motor adaptation (Haith and Krakauer, 2013). When the dynamic perturbation is removed after adaptation and the subject is reaching under unperturbed conditions, hand trajectories are deviated again. Now, the hand trajectories show after-effects in a direction opposite to the initial deviation of the dynamic perturbation. This is taken as evidence that the sensorimotor system learned an internal model to specifically counteract the dynamic perturbation and did not simply increase arm stiffness (Shadmehr et al., 2010). To detect adaptation of such an internal model, usually error clamp trials or catch trials are interspersed. In error clamp trials, movements are constrained to a virtual channel and the subjects' forces against the channel wall are evaluated (Scheidt et al., 2000). In catch trials, the dynamic perturbation is randomly and without prior announcement removed (usually in 10–20% of the trials) and subjects reach under null field conditions. This allows detection of after-effects (Brashers-Krug et al., 1996; Shadmehr and Brashers-Krug, 1997). In contrast to error clamp trials, catch trials produce large motor errors that are fed back to the subject. As motor adaptation from one trial to the next was shown to be proportional to experienced motor error (Thoroughman and Shadmehr, 2000; Donchin et al., 2003), it is widely accepted that catch trials affect execution of immediately following movement trials (Thoroughman and Shadmehr, 2000; Scheidt et al., 2001; Karniel and Mussa-Ivaldi, 2002, 2003; Levy et al., 2010). However, the influence of catch trials on the overall motor adaptation process has not yet been investigated in detail.

Following adaptation, motor memory is transformed from an initially fragile state to a more robust and stable state and therewith gains resistance to interference. This time-dependent process is called consolidation and contributes to enhanced retest performance when exposed to the disturbance a second time (Robertson et al., 2004; Krakauer and Shadmehr, 2006). In the context of force field experiments, numerous studies were able to detect enhanced retest performance of a learned force field A when exposed to this perturbation a second time (savings in AA-paradigm) (Brashers-Krug et al., 1996; Shadmehr and Brashers-Krug, 1997; Caithness et al., 2004; Overduin et al., 2006; Focke et al., 2013). Moreover, various studies investigated the consolidation process of force field adaptation using an ABA-paradigm. Thereby, consolidation following adaptation to force field A is interfered by learning a second force field B before retest of A (Brashers-Krug et al., 1996; Shadmehr and Brashers-Krug, 1997; Caithness et al., 2004; Focke et al., 2013). Some researchers found evidence for consolidation of force field A (Brashers-Krug et al., 1996; Shadmehr and Brashers-Krug, 1997), whereas others did not (Caithness et al., 2004; Focke et al., 2013). Most of these studies used catch trials without taking into account that these change the conditions of practice and may thus considerably influence the consolidation process. Indeed, Overduin et al. (2006) showed that subjects are able to consolidate a learned force field A in the ABA-paradigm when catch trials were interspersed during adaptation, whereas learning without catch trials did not lead to consolidation of force field A. Conversely, Focke et al. (2013) failed to confirm this finding for a more complex task, suggesting that not the presence of catch trials *per se* but the amount of induced catch trials might be crucial. Thus, consolidation also seems to be a practice-dependent process in which the effect of catch trials is insufficiently understood and needs to be further investigated.

Taken together, the detailed influence of catch trials on the overall motor adaptation process as well as on the following consolidation process remains unknown. Research in skill learning exhibited that variable practice schedules facilitate consolidation when learning closed tasks for which the environmental conditions are always similar and the movement can be planned in advance (Shea and Morgan, 1979; Shea and Kohl, 1991; Schmidt and Lee, 2011). Thereby, higher variability during practice leads to a poorer performance during learning but to a better performance at retest compared to lower variability during practice. Although, the relationship between motor adaptation and skill learning is far from clear (Yarrow et al., 2009), similar results may occur for motor adaptation and the following consolidation process. Therefore, the aim of our study was to investigate the influence of different catch trial ratios both on the motor adaptation process and on the consolidation process in force field adaptation. We hypothesized that increasing intermittence during practice—operationalized with various catch trial ratios of up to 40%—leads to a poorer performance during adaptation compared to lower intermittence during practice (e.g., 0% catch trials) but facilitates the consolidation process.

## Materials and methods

### Subjects

A total of 110 healthy subjects participated in this study (age 24.3 ± 2.1 years; 46 female, 64 male; 103 right-handed, 7 left-handed). They all gave written informed consent and the test-protocol was reviewed and approved by the institutional review board. All subjects were naive to the experimental procedure (apparatus, paradigm, and purpose of the study) and had no known motor deficits or neurological impairments. Handedness was verified using Edinburgh Handedness Inventory (Oldfield, 1971).

The subjects were randomly assigned to ten groups, whereas five control groups (C0, C10, C20, C30, C40) and five corresponding test groups (T0, T10, T20, T30, T40) were defined (Table 1). To investigate consolidation patterns of motor memory, we considered all ten groups separately. To analyze motor adaptation to force field A during the learning session (day 1), we unified each two corresponding groups (e.g., C10 and T10) as the corresponding control and test groups passed the same experimental procedure on that day. We refer to the union of two such groups as the catch trial ratio groups 0, 10, 20, 30, and 40%.

**Table 1:** **Experimental paradigm and classification of subject groups**.

**Group**		**Catch trial ratio (%)**	**Subjects**	**Paradigm**		
				**Day 1 (Learning)**	**Day 2 (Interference)**	**Day 3 (Retest)**
Control 0%	(C0)	0	*n* = 11	F N A_0_	–	A_0_
Test 0%	(T0)		*n* = 11	F N A_0_	B_0_ = −A_0_	A_0_
Control 10%	(C10)	10	*n* = 11	F N A_10_	–	A_10_
Test 10%	(T10)		*n* = 10	F N A_10_	B_10_ = −A_10_	A_10_
Control 20%	(C20)	20	*n* = 11	F N A_20_	–	A_20_
Test 20%	(T20)		*n* = 9	F N A_20_	B_20_ = −A_20_	A_20_
Control 30%	(C30)	30	*n* = 11	F N A_30_	–	A_30_
Test 30%	(T30)		*n* = 10	F N A_30_	B_30_ = −A_30_	A_30_
Control 40%	(C40)	40	*n* = 10	F N A_40_	–	A_40_
Test 40%	(T40)		*n* = 11	F N A_40_	B_40_ = −A_40_	A_40_

*F, familiarization block in null field (25 sets, 400 trials); N, baseline block in null field (6 sets, 96 trials); A, clockwise velocity-dependent force field with different ratio of catch trials and force field trials (25 sets, 400 trials); B, counterclockwise velocity-dependent force field with different ratio of catch trials and force field trials (25 sets, 400 trials)*.

Five subjects were excluded from the analysis because of technical reasons or lacking ability to adapt to the dynamics.

### Apparatus

**Figure 1:**
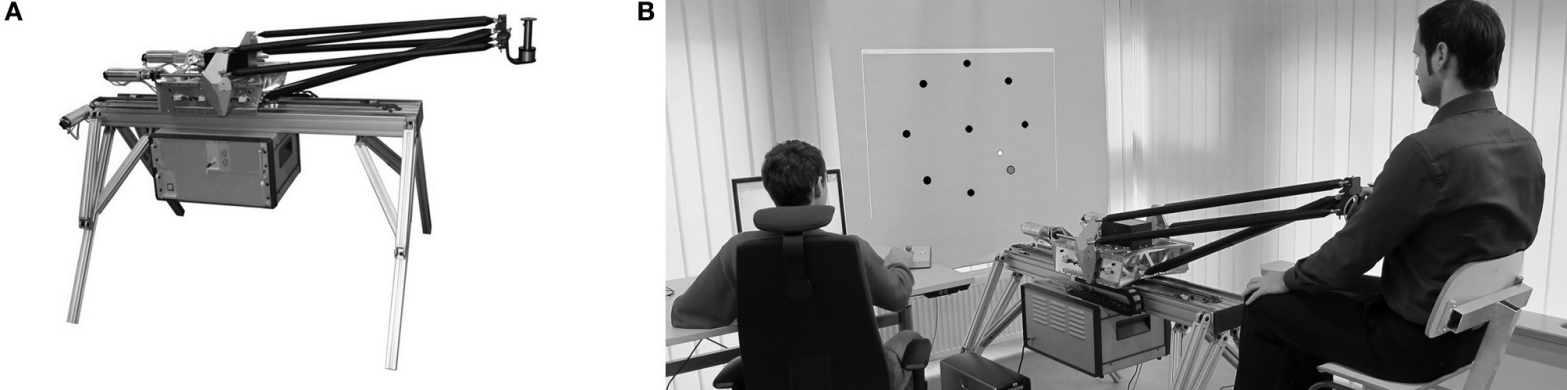
**(A)** Robotic device BioMotionBot. **(B)** Subject performing the horizontal point-to-point reaching task. The cursor corresponding to the position of the handle and the targets were displayed on a screen facing the subject.

Subjects grasped the handle of a robotic device (BioMotionBot; Figure 1A) that could exert forces (Bartenbach et al., 2013). The subjects' arms were not supported and all movements were restricted to the horizontal plane. Subjects had clear view of their hand throughout the whole experiment. They received full visual feedback of the targets as well as of the cursor corresponding to the position of the handle on a vertical screen mounted above the robotic device. Subjects sat on a chair, which was individually adjusted so that they were able to grasp the handle with their dominant hand and comfortable reach all target positions (Figure 1B). This individual seating position was reinstated in all following practice sessions. Position and force at the handle were recorded at a sampling rate of 200 Hz.

### Procedure

#### Task

We used an experimental setup similar to that described by Focke et al. (2013). Subjects were asked to perform accurate goal-directed point-to-point reaching movements in the horizontal plane with their dominant hand using the robotic device. Starting from a center point, subjects had to reach for one of the eight peripheral target points which highlighted in a pseudo-randomized order. The subsequent movement was initiated from this point back toward the center point. Therefore, the end point of each movement was the starting point for the subsequent movement. The peripheral target points were uniformly arranged on a circle of 10 cm radius around the center point. Targets were red circles (1 cm diameter) and the cursor was a white circle (0.3 cm diameter) appearing on a black background. If a target had to be reached, it changed its color from red to yellow. To avoid target sequence specific phenomena, the target sequence differed for each subject.

We defined a set of movements as 16 trials—eight outward and eight inward movements—in which each peripheral target point occurred exactly once. All learning blocks were constructed as concatenation of such movement sets. This ensured the same amount of practice toward each target direction.

Subjects were requested to perform each movement within 500 ± 50 ms. Additionally, subjects were told that reaction time was not important, i.e., after appearance of the new target they could wait as long as they wanted before starting the movement. Consequently, reaction time was excluded from the requested time interval. After completion of each movement, subjects received visual feedback about movement time on the screen. If the subjects reached the target within the required time, a green circle around the target appeared. If they moved too slowly, a red circle appeared and when moving too fast, an orange circle turned up. This visual feedback was provided throughout the whole experiment to ensure consistent movement speed.

#### Experimental design

To investigate the consolidation process of a learned task A, we used an ABA-paradigm whereby the practice sessions were distributed over three days with 24 h rest between each session (Table 1). To determine the adaptation process, we considered the learning block A on day 1.

On day 1, all subjects began with a familiarization block under null field conditions (F, no disturbing forces) for 25 sets (400 trials; Table 1). After performing this familiarization block, subjects were able to perform the movements at the requested speed. We did not further analyze this data. After 5 min of rest, subjects performed a baseline block for six sets (96 trials) under null field conditions (N). Based on these trials, we calculated baseline trajectories to evaluate null field performance and as reference to movement trials performed under force field conditions. After another 5 min of rest, subjects performed 25 sets (400 trials) in a velocity-dependent clockwise force field A. On day 2 (24 h rest), subjects of the test groups (T0, …, T40) were exposed to a second interfering velocity-dependent counterclockwise force field B = −A for 25 sets. Subjects of the control groups did not attend the laboratory on day 2. On day 3, all subjects were retested for another 25 sets in force field A.

During force field adaptation, short breaks of 60 s were inserted after each five sets. Thereby, subjects could release their hand from the handle but remained seated. The sessions lasted approximately 60 min on day 1 and approximately 30 min on the subsequent practice days. Subjects were instructed to sleep at least 6 h between the test sessions.

#### Forces and catch trials

Within the force field adaptation blocks, the robotic device generated a velocity-dependent force field that applied forces perpendicular to the direction of movement according to the following equation:

$$\left[\begin{array}{c} F_{x}\\ F_{z} \end{array}\right]=k\cdot \left[\begin{array}{cc} \cos(\theta ) & -\sin(\theta )\\ \sin(\theta ) & \cos(\theta ) \end{array}\right]\cdot \left[\begin{array}{c} \dot{x}\\ \dot{z} \end{array}\right]$$

Here, *F_x_* and *F_z_* are the robot-generated forces; *k* = 20 Ns/m is the force field viscosity; θ denotes the direction of the force field (force field A: θ = −90° clockwise-directed, force field B: θ = 90° counterclockwise-directed); *ẋ* and *ż* are the components of hand velocity in the horizontal plane.

During force field adaptation, catch trials were pseudo-randomly interspersed without prior announcement. Depending on the group, catch trials appeared with either 0, 10, 20, 30, or 40% probability. The catch trial ratio is indexed in the name of group and applied force field, respectively (e.g., C10: control group adapting to force field A_10_ with 10% catch trials; Table 1). Catch trials occurred in outward and inward movements and in some cases occurred one after another. If a catch trial occurred toward a specific direction, no force field trial was performed toward this direction during this set of movements. Catch trials were induced without replacement such that the number of force field trials differed between the catch trial groups but the total amount of performed movements retained (400 movements).

### Data analysis

#### Preprocessing

All parameters were calculated using the custom-made software application ManipAnalysis (Stockinger et al., 2012). Raw data of hand trajectories were filtered using a fourth-order Butterworth low-pass filter (6 Hz cut-off frequency). Afterwards, movement velocities were numerically computed using central difference method. Next, data sets were segmented. For position data, movement start was defined as the time-point when the cursor left the starting point and movement termination was marked when the cursor reached the target point. For velocity data, movement onset (or end) was defined as the time at which velocity exceeded (or fell under) 10% of maximal velocity of that movement. Finally, the data sets were time-normalized using cubic spline interpolation to make them comparable.

We calculated baseline trajectories and baseline velocity profiles for each of the 16 movement directions by respectively averaging corresponding movements of the last five sets recorded in the baseline block under null field conditions (N) (Stockinger et al., 2012).

#### Performance measurement

***Velocity vector correlation coefficient***. To quantify movement performance under force field conditions, we calculated a velocity vector correlation coefficient. This widely used measure only considers force field trials and quantifies motor performance by estimating the similarity between the velocity profiles of force field movements and corresponding baseline movements (Shadmehr and Brashers-Krug, 1997; Caithness et al., 2004; Overduin et al., 2006; Stockinger et al., 2012).

***Perpendicular displacement***. To specifically evaluate catch trial movements, we calculated the signed perpendicular displacement (PD_catch_) of hand trajectory from the straight line joining start and target point 300 ms after movement start (Shadmehr and Brashers-Krug, 1997; Donchin et al., 2002). This measure allowed us to gauge both the magnitude and the direction of the deviation. For instance, a subject who adapted to a clockwise-directed force field A will predictively generate additional forces in counterclockwise direction to cancel out the expected disturbing forces (Shadmehr and Mussa-Ivaldi, 1994). Consequently, we would expect the perpendicular displacement on a catch trial to be counterclockwise-directed. We indicate such after-effects appropriate to force field A with negative sign. In contrast, we indicate after-effects appropriate to force field B with positive sign.

Moreover, the perpendicular displacement was calculated for force field trials (PD_field_) to calculate a learning index as described in the following paragraph. Other measures of trajectory displacement (e.g., maximal perpendicular displacement, mean perpendicular displacement, perpendicular displacement 200 ms after movement start) yielded qualitatively similar results and are therefore not presented in this paper.

***Learning index***. To relate force field trials and catch trials, we calculated a learning index (LI). This learning index allows quantification of force field learning with respect to after-effects during catch trials (Donchin et al., 2002; Overduin et al., 2006). When subjects adapt to the force field conditions, trajectories should become straight-lined and therefore show gradually decreasing perpendicular displacement values in force field trials. However, in catch trials there should be increasing after-effects to the opposite direction with ongoing learning (Shadmehr and Mussa-Ivaldi, 1994). Based on this idea we calculated the learning index as follows:

$$\text{LI}=\;\frac{{\bar{\text{PD}}}_{\text{catch}}}{\left|{\bar{\text{PD}}}_{\text{field}}\right|+\left|{\bar{\text{PD}}}_{\text{catch}}\right|}\in [-1,1]$$

Thereby, PD denotes the perpendicular displacement of hand trajectory as defined above in either force field trials (PD_field_) or catch trials (PD_catch_). The learning index was calculated using perpendicular displacement mean values (PD) of force field and catch trials for each set (16 trials) of movements.

Early in the adaptation period, subjects should show a learning index near zero because in catch trials small after-effects and in force field trials large displacements should appear. With ongoing practice, the absolute value of the learning index should increase because after-effects in catch trials increase and deviations in force field trials decrease (Donchin et al., 2002). A subject who resists the disturbing forces by increasing the stiffness of the arm may perform an accurate movement showing only small deviations during force field trials. Nevertheless, this leads to a low-valued learning index because perpendicular displacements are also small in catch trials. Thus, the learning index is a good measure to quantify force field prediction and thus internal model formation (Overduin et al., 2006).

The learning index is a relative measure of performance with a theoretical limit of 1 (absolute value). It is signed as the numerator includes the signed perpendicular displacement of catch trials. This allows distinction of learning the two opposing force fields A and B. Thereby, learning of the clockwise-directed force field A was indicated with a negative value, whereas learning of the counterclockwise-directed force field B had positive sign.

The learning index was not calculated for the 0% catch trial groups (C0, T0) as these groups did not receive any catch trials that indicate after-effects.

We conducted the statistical analyses for velocity vector correlation coefficient, learning index as well as the perpendicular displacement. We did this for several reasons. First, velocity vector correlation is a well-established and frequently used measure to quantify force field learning (e.g., Shadmehr and Brashers-Krug, 1997; Caithness et al., 2004; Overduin et al., 2006). This enables comparison to most former force field studies. Second, velocity vector correlation allowed us to quantify performance of the 0% catch trial group which is impossible when using the learning index. Third, we additionally used the learning index based on the perpendicular displacement because it also considers catch trials and therefore more emphasizes the internal model prediction and the direction of force prediction than the velocity vector correlation. Furthermore, the perpendicular displacement is an intuitively accessible and frequently used measure of motor error (e.g., Shadmehr and Brashers-Krug, 1997; Donchin et al., 2002).

***Difference values***. To assess performance changes between two distinct points in time, we calculated the difference value of performances between these two points in time. Thereby, increase (or decrease) of performance within the considered period was indicated with positive (or negative) sign. Using this difference value we were able to compare performance changes across different groups.

The term “initial performance” always refers to the mean score of the first set of movements (16 trials) within the considered period. Accordingly, “end performance” always refers to the mean score of the last set of movements (16 trials) within the considered period.

#### Statistics

To test for differences within groups, we used paired *t*-tests. Adaptation on day 1 was confirmed by comparing initial and end performance of the learning session for each group. To check for consolidation of force field A of a specific group, we compared initial performance of the learning session (day 1) and retest session (day 3) of that group.

To test for differences between groups, we conducted One-Way ANOVAs with between subject factor group. Hereby, differences in initial or end performance were determined. To compare the degree of adaptation between groups, we considered the difference value of initial and end performance in force field A of the learning session (day 1).

To test for differences in consolidation between the pairs of groups, we conducted a Two-Way ANOVA with the between subject factors catch trial ratio (0, 10, 20, 30, 40%) and interference (control group, test group). Therefore, we compared the difference values calculated from initial performance of the learning session (day 1) and retest session (day 3) between the different groups. This allowed evaluation of the influence of different catch trial ratios on the consolidation of force field A with respect to the interference of force field B.

To evaluate after-effects, we used one-sample *t*-tests to compare given mean values to zero.

All statistical analyses were conducted using IBM SPSS software (v.21). All data are presented as mean values and 95% confidence intervals. For all statistical tests, the level of significance was a priori set to *p* = 0.05. If One-Way ANOVAs revealed significant differences, Bonferroni *Post-hoc* analysis was used. Effect sizes were determined using partial eta squared η^2^_*p*_ (small effect: η^2^_*p*_ = 0.01; medium effect: η^2^_*p*_ = 0.06; large effect: η^2^_*p*_ = 0.14) or Cohen's *d* (small effect: *d* = 0.20; medium effect: *d* = 0.50, high effect: *d* = 0.80) (Cohen, 1992). All correlation coefficients were transformed using Fisher *z*-transform before statistical analyses were conducted. All presented data of velocity vector correlation refers to the retransformed *z*-values.

## Results

The initial performance of the learning session (day 1, first set) did not differ significantly between the ten groups (One-Way ANOVA, factor: group (C0, …, C40, T0, …, T40)]. This holds for the velocity vector correlation [*F*_(9, 95)_ = 1.30, *p* = 0.249], perpendicular displacement [*F*_(9, 95)_ = 1.58, *p* = 0.135], and learning index [*F*_(7, 75)_ = 0.92, *p* = 0.493]. Furthermore, we found no significant differences in initial (first set) or end performance (last set) between corresponding control and test groups which received the same amount of catch trials (pairwise independent *t*-tests). These findings hold for velocity vector correlation, perpendicular displacement, and learning index. Thus, we can unify corresponding control and test groups to analyze the adaptation process during the learning session (day 1) as subjects received the same protocol on that day. Similarly to the ten separated groups, we found no significant differences in initial performance of the learning session (day 1) when considering the five different catch trial ratio groups (One-Way ANOVA, factor: catch trial ratio (0%, …, 40%); unification of each two corresponding control and test groups) for velocity vector correlation [*F*_(4, 100)_ = 1.42, *p* = 0.234], perpendicular displacement [*F*_(4, 100)_ = 1.42, *p* = 0.222], and learning index [*F*_(3, 79)_ = 0.68, *p* = 0.568]. Therefore, we act on the assumption that all groups and all catch trial ratio groups started with similar initial conditions.

In the following, we first present results of the adaptation process of the learning session (day 1) considering the five unified catch trial ratio groups. Afterwards, we present results of the consolidation of force field A. The test groups were exposed to a second interfering force field B on day 2 before retest of force field A on day 3, whereas the control groups were retested in force field A on day 3 without interference. Therefore, in consolidation analysis, we consider all ten control and test groups separately (Table 1). Additionally, we present results of the after-effects detected during catch trials.

### Adaptation

#### Hand trajectories and velocity profiles

**Figure 2:**
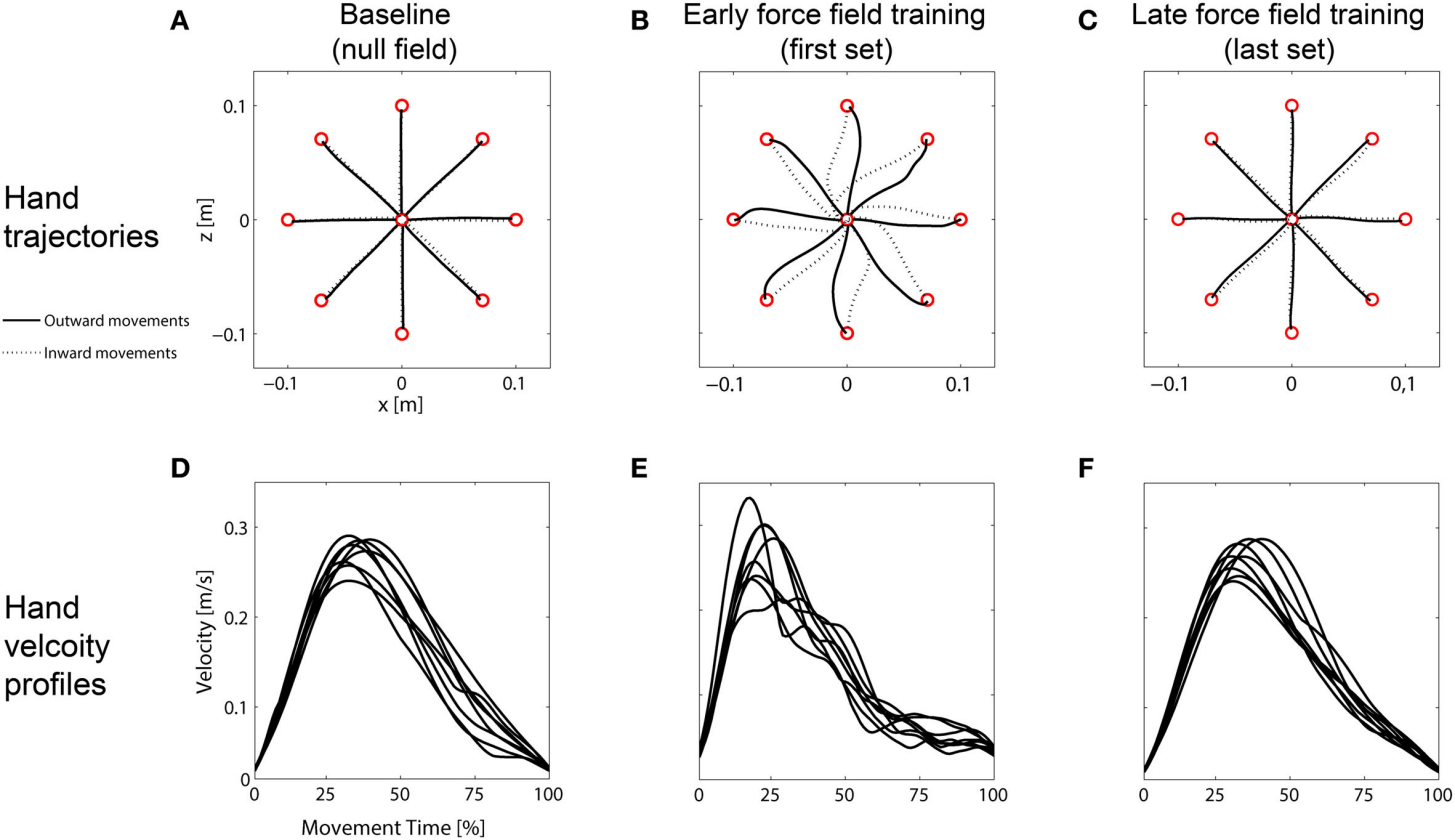
**Representative mean hand trajectories and mean hand velocity profiles (outward movements only) of one group. (A)** Straight-lined baseline trajectories. **(B)** Disturbed trajectories at the beginning of force field adaptation (first set). **(C)** Reshaped straight-lined trajectories at the end of force field adaptation (last set). **(D)** Smooth bell-shaped, single-peak baseline velocity profiles. **(E)** Disturbed velocity profiles at the beginning of force field adaptation. **(F)** Velocity profiles at the end of force field adaptation showing bell-shaped, single-peak profiles.

On day 1, all subjects showed the expected adaptation pattern when exposed to force field A. At the beginning of force field adaptation, subjects' hands were considerably disturbed. This resulted in distinctively curved trajectories compared to the null field condition (Figures 2A,B). The force field disturbance was also indicated by a change in the hand velocity profiles. Under null field conditions, subjects produced typical bell-shaped velocity profiles with a single peak (Figure 2D). At the beginning of force field adaptation, however, subjects' velocity profiles were noticeably disturbed (Figure 2E). With practice, subjects were able to counteract the forces resulting in straight-lined trajectories and velocity profiles similar to those profiles in baseline movements (Figures 2C,F).

#### Velocity vector correlation coefficient and perpendicular displacement

**Figure 3:**
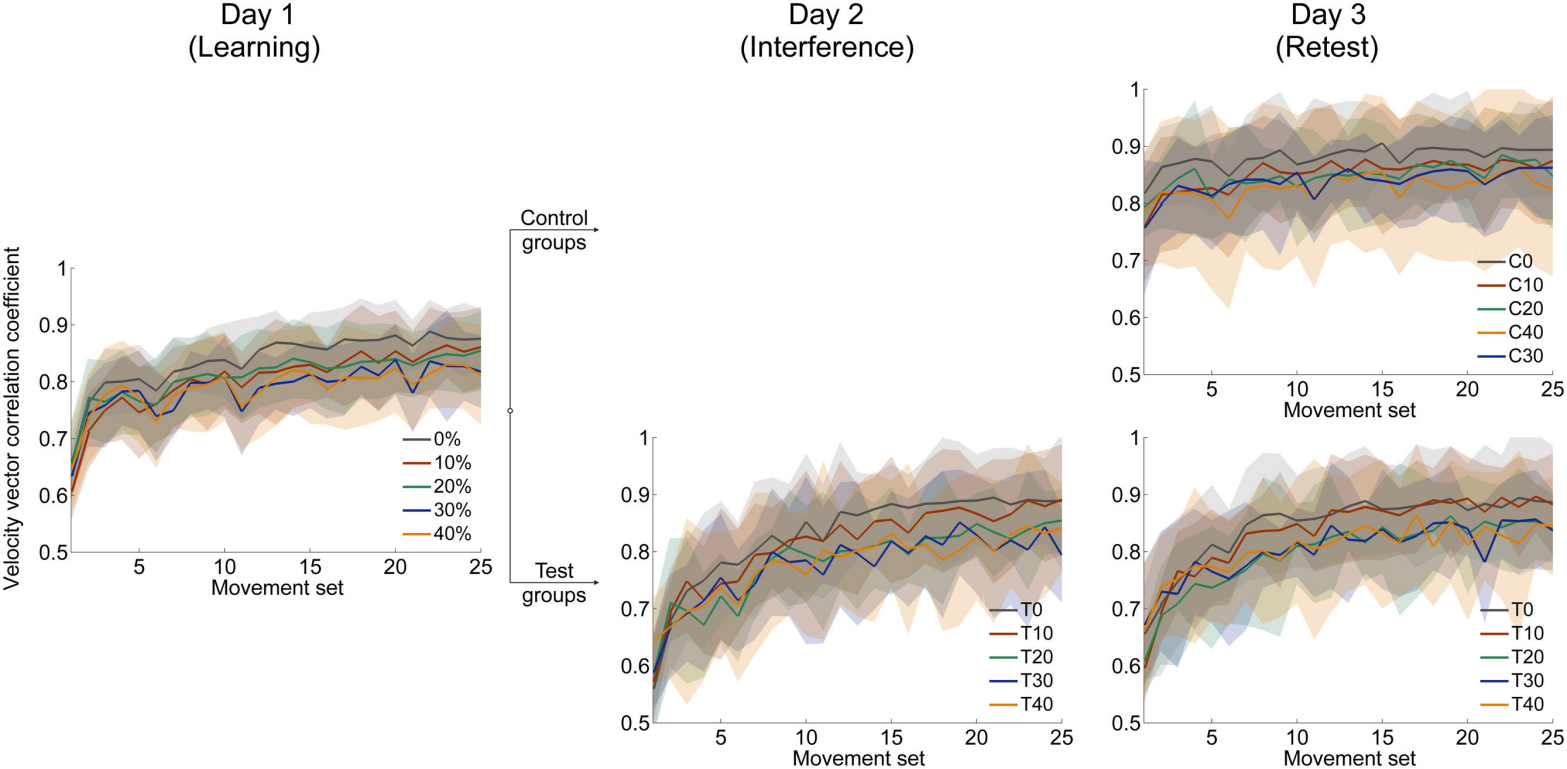
**Mean time courses of velocity vector correlation coefficient for all three days**. After learning force field A on day 1 **(left)**, subjects of each catch trial ratio group were divided into control and test groups. Test groups adapted to an interfering force field B = −A on day 2 **(mid)**. On day 3, all groups were retested in force field A **(right)**. On all three days, subjects were able to adapt to the changed dynamic conditions indicated by increasing correlation coefficients. All data is presented as mean values ±95% confidence intervals.

**Figure 4:**
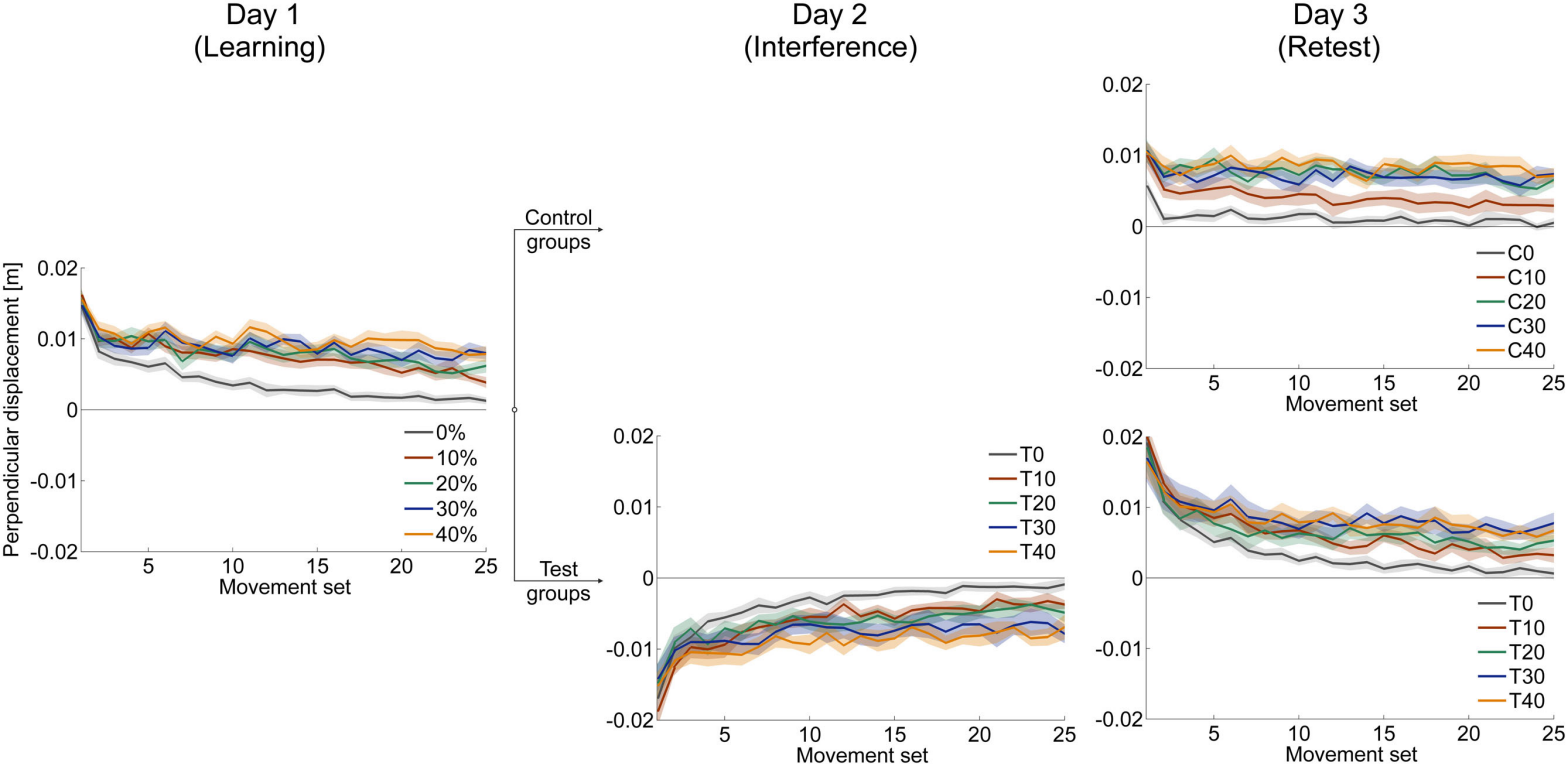
**Mean time courses of signed perpendicular displacement 300 ms after movement start in force field trials**. Positive (negative) values indicate deviations in clockwise (counterclockwise) direction caused by disturbance of force field A (force field B). On all three days, subjects were able to adapt to the changed dynamic conditions leading to decreased errors. All data is presented as mean values ±95% confidence intervals.

The time course of velocity vector correlation coefficient and perpendicular displacement demonstrate the progress of adaptation to force field A for all groups (Figure 3, left; Figure 4, left). Adaptation is illustrated by a distinct improvement of motor performance during force field learning. Initially, all groups show rapid improvements in performance. With further practice, the rate of performance improvement decreases. Finally, performance output reaches plateau.

We statistically confirmed adaptation to force field A for each catch trial ratio group. Thereby, all groups showed significantly higher end performance compared to the initial performance (paired *t*-test: *p* < 0.001 for all five groups). Moreover, we found differences in the degree of adaptation between the five catch trial ratio groups: The performance improvement value assessed by subtracting initial performance from end performance showed significant differences between the five groups. All these findings hold for both velocity vector correlation [One-Way ANOVA: *F*_(4, 100)_ = 6.70, *p* < 0.001; Figure 7A] and perpendicular displacement [One-Way ANOVA: *F*_(4, 100)_ = 24.64, *p* < 0.001; Figure 7B]. For the velocity vector correlation, Bonferroni *Post-hoc* analysis revealed significant differences between 0 and 30% catch trial ratio (*p* = 0.015), 0 and 40% catch trial ratio (*p* = 0.001), 10 and 30% catch trial ratio (*p* = 0.015), as well as between 10 and 40% catch trial ratio (*p* = 0.015). For the perpendicular displacement, Bonferroni *Post-hoc* analysis revealed significant differences between 0 and 20% catch trial ratio (*p* < 0.001), 0 and 30% catch trial ratio (*p* < 0.001), 0 and 40% catch trial ratio (*p* < 0.001), 10 and 20% catch trial ratio (*p* = 0.002), 10 and 30% catch trial ratio (*p* < 0.001), 10 and 40% catch trial ratio (*p* < 0.001), as well as between 20 and 40% catch trial ratio (*p* = 0.024).

Additionally, we conducted the same analyses comparing the performance at the time point at which all groups had performed a total of 240 force field trials, i.e., same amount of force field trials. We found similar results, i.e., significant worse degree of adaptation with increasing catch trial ratio for velocity vector correlation [One-Way ANOVA: *F*_(4, 100)_ = 3.31, *p* = 0.014] and perpendicular displacement [One-Way ANOVA: *F*_(4,100)_ = 13.35, *p* < 0.001]. Thus, the reported differences between the catch trial ratio groups were not because of the different amount of performed force field trials. Therefore, sensorimotor adaptation, as quantified by the velocity vector correlation coefficient and perpendicular displacement, worsened as the catch trial ratio increased.

#### Learning index

To determine the degree of force field learning with respect to catch trials, we conducted the same adaptation analyses as above for the four groups that received catch trials using the learning index. All four groups improved rapidly at the beginning of adaptation (Figure 6, left). This rapid improvement decayed with ongoing practice and finally reached plateau for all groups. All groups were able to adapt to the force field conditions when considering the learning index. This was exhibited by a significant improvement from first set to last set (paired *t*-test: *p* < 0.001 for all four groups).

To gauge differences in the improvement of learning index during adaptation, we compared the difference values (initial vs. end of adaptation) of learning index and found significant differences between the four groups [One-Way ANOVA: *F*_(3, 79)_ = 2.85, *p* = 0.043]. Bonferroni *Post-hoc* analysis revealed significant differences between 10 and 30% catch trial ratio (*p* = 0.032) as well as between 10 and 40% catch trial ratio (*p* = 0.016) (Figure 7C). These differences also hold, when evaluating the learning index at the time point at which all groups had performed 240 force field trials [One-Way ANOVA: *F*_(3, 79)_ = 3.53, *p* = 0.019]. Therefore, sensorimotor adaptation, as quantified by the learning index, worsened as the catch trial ratio increased. These results are in accordance to the results of velocity vector correlation and perpendicular displacement mentioned above.

#### Adaptation to the interfering force field

On day 2, all test groups were exposed to the interfering force field B and followed the same protocol as on day 1, respectively. Thus, these groups received different amounts of catch trials. We found differences in the degree of adaptation to force field B between the test groups: The performance improvement value assessed by subtracting initial performance from end performance showed significant differences between the five test groups for all measures [One-Way ANOVA: velocity vector correlation: *F*_(4, 46)_ = 7.564, *p* < 0.001; perpendicular displacement: *F*_(4, 46)_ = 11.407, *p* < 0.001; learning index: *F*_(3, 36)_ = 7.561, *p* < 0.001]. For the velocity vector correlation, Bonferroni *Post-hoc* analysis revealed significant differences between T0 and T30 (*p* = 0.001), T0 and T40 (*p* = 0.007), T10 and T30 (*p* = 0.002), as well as between T10 and T40 (*p* = 0.009). For the perpendicular displacement, Bonferroni *Post-hoc* analysis revealed significant differences between T0 and T20 (*p* = 0.019), T0 and T30 (*p* < 0.001), T0 and T40 (*p* = 0.001), T10 and T30 (*p* < 0.001), as well as between T10 and T40 (*p* = 0.006). For the learning index, Bonferroni *Post-hoc* analysis revealed significant differences between T10 and T30 (*p* = 0.001) and between T10 and T40 (*p* = 0.001). Therefore, the test groups' attained level of adaptation to force field B decreased with increasing catch trial ratio which is in line with the findings on the adaptation to force field A on day 1.

### Consolidation

#### Velocity vector correlation coefficient and perpendicular displacement

As expected, all control groups exhibited savings of force field A from learning on day 1 until retest on day 3. This was indicated by a significant increase of the initial performance (mean score of first set) measured by velocity vector correlation from learning session (day 1) to retest (day 3) of force field A for all control groups (paired *t*-test: *p* < 0.010 for all five groups). In particular, we found no significant differences in this increase between the five control groups [One-Way ANOVA: *F*_(4, 49)_ = 2.31, *p* = 0.073] when comparing the difference value of initial performance on day 1 and day 3, i.e., the consolidation processed similarly for all control groups.

The aim of our study was to investigate the influence of different catch trial ratios on the consolidation process in the ABA-paradigm, in particular on resistance to interference of force field B learning. To assess the influence of catch trials on the consolidation process with respect to the interference of force field B, we conducted a Two-Way ANOVA [between subject factors: catch trial ratio (0, …, 40%) and interference (control group, test group)] analyzing the difference values of initial performances of the learning session and retest of each catch trial ratio group (Figure 8A). We found a significant interference effect [*F*_(1, 95)_ = 65.90, *p* < 0.001, η^2^_*p*_ = 0.41] and a significant interaction between interference and catch trial ratio [*F*_(4, 95)_ = 5.11, *p* = 0.001, η^2^_*p*_ = 0.18] Thus, in general, exposure to the interfering force field B on day 2 had an effect on the consolidation process. However, for different catch trial ratio groups, consolidation progresses differently. *Post-hoc* analysis (pairwise independent *t-tests* between corresponding control and test groups) revealed significant differences between groups C0 and T0 [*t*_(20)_ = 5.79, *p* < 0.001, *d* = 2.47], C10 and T10 [*t*_(19)_ = 4.68, *p* < 0.001, *d* = 2.06], C20 and T20 [*t*_(18)_ = 6.27, *p* < 0.001, *d* = 2.85] as well as between C40 and T40 [*t*_(19)_ = 2.74, *p* = 0.013, *d* = 1.18] indicating a significant effect of the exposure to the interfering force field B, respectively. However, we found no significant differences between C30 and T30 [*t*_(19)_ = −0.02, *p* = 0.983, *d* = 0.01]. Thus, the consolidation process of subjects receiving 30% catch trials was not significantly influenced by the interfering force field B when measured by velocity vector correlation. Taken together, for the catch trial ratio groups 0, 10, and 20% the difference values comparing initial performance of the learning session (day 1) and retest (day 3) differed significantly between control and test groups. Thereby, test groups performed worse. This significant difference did not appear for a catch trial ratio of 30%. In case of a further increase of the catch trial ratio up to 40%, control and test groups showed significant differences again.

We conducted the same analyses for the performance values assessed by the perpendicular displacement (Figure 8B). The Two-Way ANOVA (between subject factors: catch trial ratio [0, …, 40%] and interference (control group, test group)] analyzing the difference values of initial performances of the learning session and retest revealed a significant interference effect [*F*_(1, 95)_ = 179.21, *p* < 0.001, η^2^_*p*_ = 0.65] and a significant interaction between interference and catch trial ratio [*F*_(4, 95)_ = 3.10, *p* = 0.019, η^2^_*p*_ = 0.12]. In general, this indicates an effect of exposure to force field B and differing consolidation progresses for the diverse catch trial ratios which is in line with the findings of the velocity vector correlation. In contrast, *Post-hoc* analysis (pairwise independent *t*-tests), showed significant differences between all corresponding control and test groups, respectively [0%: *t*_(20)_ = 7.96, *p* < 0.001, *d* = 3.39; 10%: *t*_(19)_ = 7.13, *p* < 0.001, *d* = 3.12; 20%: *t*_(18)_ = 6.10, *p* < 0.001, *d* = 2.71; 30%: *t*_(19)_ = 4.28, *p* < 0.001, *d* = 1.86; 40%: *t*_(19)_ = 4.49, *p* < 0.001, *d* = 1.97]. Therefore, exposure to force field B significantly impaired the consolidation process of all test groups. However, this impairment depended on the catch trial ratio and was least for the 30% catch trial ratio test group compared to its control group (Figure 8B).

Thus, the consolidation process, as measured by the velocity vector correlation, was influenced by catch trials, suggesting most resistance to interference of force field B for 30% catch trials. Consolidation, in terms of absence of interference and as quantified by the perpendicular displacement, could not be detected for any catch trial ratio. However, the consolidation process was least impaired for 30% catch trial ratio suggesting partial resistance to interference.

#### Learning index

We also considered the progress of learning index from the learning session (day 1) to the retest session (day 3). All control groups showed significantly higher initial learning index values at retest than at the learning session (paired *t*-test: *p* < 0.015 for all four groups). In particular, we found no significant differences in this increase between the four control groups when comparing the difference value of initial performance on day 1 and day 3 [One-Way ANOVA: *F*_(3, 39)_ = 0.08, *p* = 0.970], i.e., the consolidation processed similarly for all control groups. This is in accordance to the findings of the velocity vector correlation and perpendicular displacement. However, the Two-Way ANOVA [between subject factors: catch trial ratio (10, …, 40%) and interference (control group, test group)] revealed only a significant effect of interference [*F*_(1, 75)_ = 129.59, *p* < 0.001, η^2^_*p*_ = 0.63; Figure 8C) but no significant interaction between interference and catch trial ratio [*F*_(3, 75)_ = 0.59, *p* = 0.625, η^2^_*p*_ = 0.02]. Therefore, exposure to force field B had a significant influence on the consolidation process for all considered catch trial ratios. Thus, in contrast to the results of velocity vector correlation and perpendicular displacement, the catch trial ratio had no significant influence on the consolidation process as quantified by the learning index.

### After-effects

**Figure 5:**
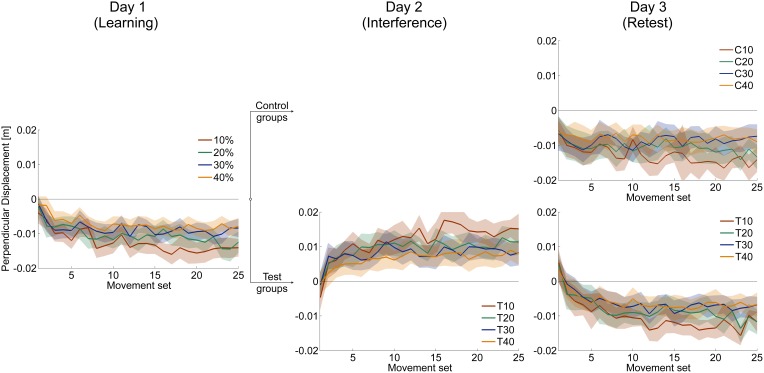
**Mean time courses of after-effects measured by signed perpendicular displacement during catch trials**. Negative (positive) values indicate deviations in counterclockwise (clockwise) direction and therefore after-effects appropriate to force field A (force field B). The magnitude of after-effects increases with ongoing practice and is least for subjects receiving 30 and 40% catch trials. All data is presented as mean values ±95% confidence intervals.

Figure 5 illustrates after-effects of force field adaptation in catch trials that occurred during all sessions. For all catch trial ratio groups, after-effects are initially small but increase with ongoing practice and reach plateau. At the end of each day, the catch trial groups' after-effects differed significantly in magnitude (One-Way ANOVAs: *p* ≤ 0.001 for all three days). Thereby, with increasing catch trial ratio, subjects showed significantly smaller after-effects.

To investigate after-effects of force field B adaptation onto retest of force field A, we considered catch trials at the beginning of the retest session (day 3). We calculated the mean values of perpendicular displacements in catch trials of the first set of movements. This allowed us to quantify the magnitude and the direction of after-effects. Comparison of after-effects based on only the first catch trial, which occurred for all groups on the third or fourth trial, lead to similar results.

At the beginning of the retest session, all control groups showed significant negative mean perpendicular displacements [one-sample *t*-test vs. zero; C10: *t*_(10)_ = −3.72, *p* = 0.004; C20: *t*_(10)_ = −3.73, *p* = 0.004; C30: *t*_(10)_ = −5.58, *p* < 0.001; C40: *t*_(9)_ = −9.38, *p* < 0.001; Figure 9]. Thus, subjects of all control groups started retest of force field A with a force field prediction appropriate to force field A. We found no significant differences of these after-effects between the control groups [One-Way ANOVA: *F*_(3, 39)_ = 0.65, *p* = 0.578].

The test groups, however, all showed significant positive perpendicular displacements in catch trials at the beginning of retest [one-sample *t*-test vs. zero; T10: *t*_(9)_ = 2.74, *p* = 0.023; T20: *t*_(8)_ = 4.87, *p* = 0.001; T30: *t*_(9)_ = 2.37, *p* = 0.042; T40: *t*_(10)_ = 2.90, *p* = 0.016; Figure 9]. Thus, subjects of all test groups started retest of force field A with a force field prediction appropriate to force field B. We found no significant differences of these after-effects between the test groups indicating similar after-effects of force field B adaptation onto retest of force field A [One-Way ANOVA: *F*_(3, 36)_ = 0.68, *p* = 0.542].

## Discussion

Our study was designed to investigate the influence of catch trials on the overall motor adaptation process as well as on the following consolidation process. We hypothesized that increasing intermittence during practice—operationalized with various catch trial ratios—leads to a poorer performance during adaptation compared to constant practice but facilitates consolidation. Against the background of these hypotheses, we separately discuss the results on motor adaptation (section Catch Trials Influence Internal Model Formation and Motor Performance During Adaptation) and consolidation (section Consolidation Depends on Catch Trial Ratio and Performance Measure). Finally, we relate our results to findings from research on skill learning (Section Comparison of Motor Adaptation and Skill Learning).

### Catch trials influence internal model formation and motor performance during adaptation

Our results on motor adaptation showed that increased intermittence by interspersed catch trials lead to poorer performance during adaptation. We assume that the catch trial induced intermittences impair the ability to form an internal model and therewith impair accurate compensation for the dynamic perturbation.

**Figure 6:**
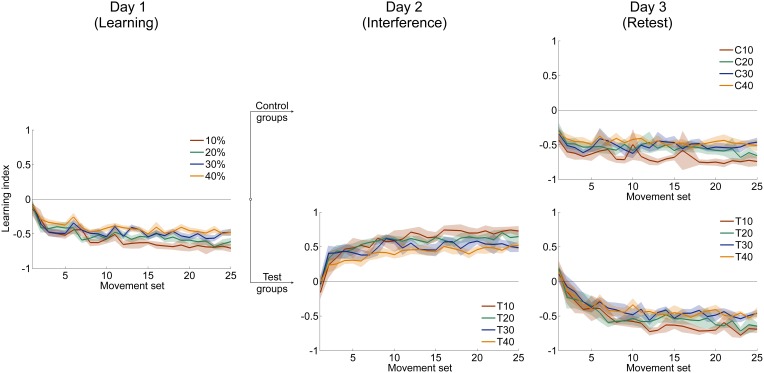
**Mean time courses of learning index which relates catch trials and force field trials**. Learning of the clockwise-directed force field A is indicated with negative values, learning of the counterclockwise-directed force field B has positive sign. All data is presented as mean values ±95% confidence intervals.

In accordance to former studies (e.g., Shadmehr and Mussa-Ivaldi, 1994), our results showed that all groups were able to adapt to the changed dynamic conditions induced by the disturbing force field (Figures 3, 4, 6). Though, the degree of adaptation depended on the amount of induced catch trials. This finding holds for all considered performance measures (Figures 3, 4, 6). Overduin et al. (2006) did not find an influence of catch trials on the performance development during adaptation. But they solely compared 0 and 20% catch trial ratio groups using the velocity vector correlation. However, we tested a wider range of catch trial ratios and performance measures demonstrating that, in general, motor adaptation depends on the catch trial ratio. These differences in the degree of adaptation are not attributed to the different amount of performed force field trials but seem to be distinctively caused by the catch trial induced intermittences.

**Figure 7:**
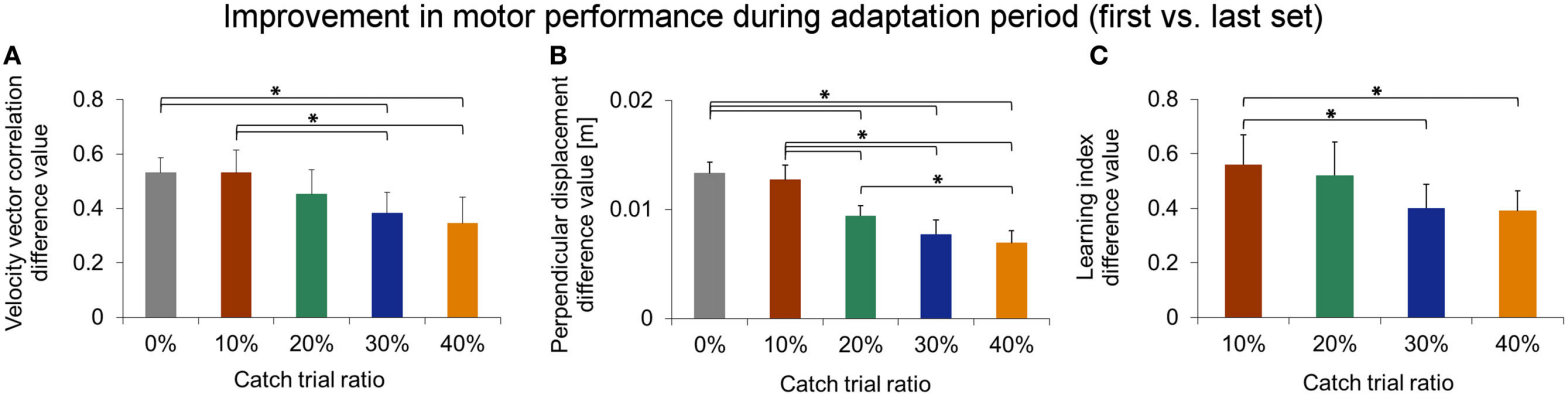
**Comparison of degree of adaptation between catch trial ratio groups using velocity vector correlation coefficient (A), perpendicular displacement (B), and learning index (C)**. All three performance measures indicate a significantly decreasing degree in force field adaptation with increasing catch trial ratio. All data is presented as mean values ±95% confidence intervals; (^*^) indicates significant differences between catch trial ratio groups.

We assume that subjects of different catch trial ratio groups had formed internal models of different quality. As mentioned above, the learning index is a good measure of force field prediction (Donchin et al., 2002; Overduin et al., 2006). Subjects receiving less catch trials (10, 20%) demonstrated learning curves that suggest better force field prediction compared to the 30% and 40% catch trial ratio groups (Figure 6, left). Accordingly, the higher learning index for low catch trial groups contributes to an appropriate internal model formation that enables accurate movement generation using a feed forward control strategy (Donchin et al., 2002; Overduin et al., 2006; Franklin et al., 2008). In contrast, the learning index values of the catch trial ratio groups 30% and 40% suggest an impaired ability to form an appropriate internal model. This would cause inaccurate prediction of perturbing forces and require reaction using muscular co-contraction to perform the movement (Overduin et al., 2006). Maybe, subjects receiving a high amount of catch trials relied more on an impedance control strategy by increasing arm stiffness (Schabowsky et al., 2007). Former research already proposed coexistence of forward model prediction and impedance control strategy (Takahashi et al., 2001; Osu et al., 2003; Milner and Franklin, 2005). Since we tested adaptation using various catch trial ratios, we propose that the ability to form an appropriate internal model changes gradually with altered conditions of practice. This might explain the order of attained performance level according to the amount of induced catch trials which is reasonable as with increasing catch trial ratio the interferences and the uncertainty increase which prevents the sensorimotor system of accurately predicting the disturbing forces (Takahashi et al., 2001; Osu et al., 2003; Franklin et al., 2008). In contrast, internal model formation is more emphasized for subjects receiving constant force field perturbations. For such constant perturbations, it was previously assumed that appropriate internal model formation is the main reason for high movement performance at the end of the learning session (Shadmehr et al., 2010).

### Consolidation depends on catch trial ratio and performance measure

**Figure 8:**
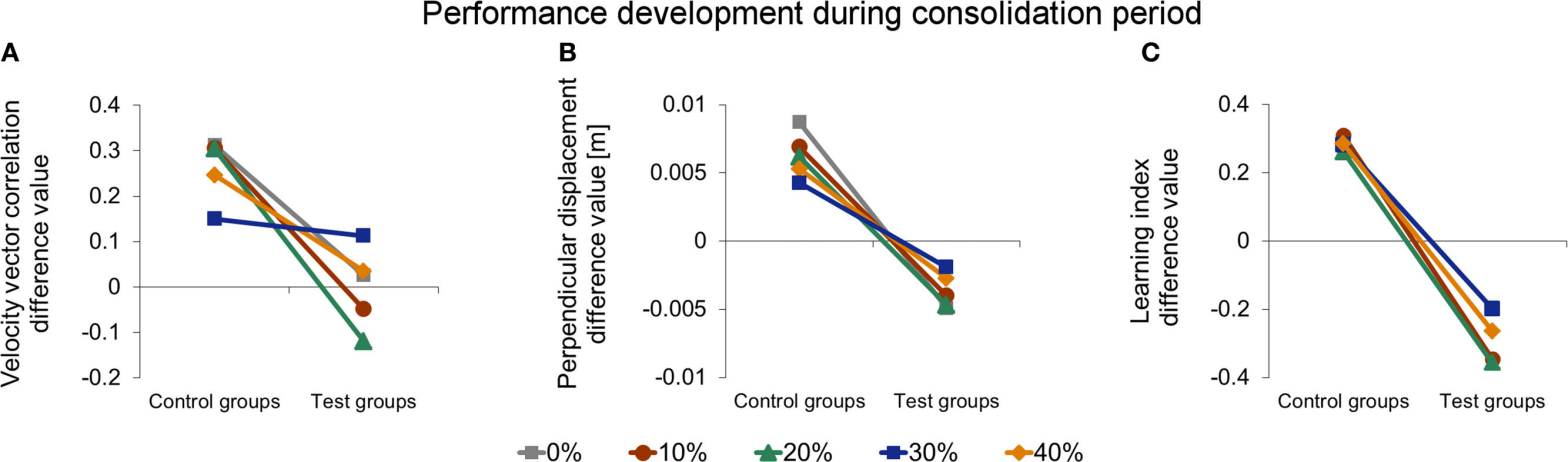
**Comparison of development in initial performance from learning session (day 1) to retest (day 3) of force field A measured by velocity vector correlation (A), perpendicular displacement (B), and learning index (C)**. Positive values indicate a performance improvement, whereas negative values indicate a decreased initial retest performance compared to naive performance. In general, test groups show impaired consolidation compared to corresponding control groups indicated by a significant effect of interference (control, test). For velocity vector correlation **(A)** and perpendicular displacement **(B)**, there is also a significant interaction of interference and catch trial ratio, indicating different consolidation depending on the catch trial ratio. Thereby, consolidation is least impaired for 30% catch trial test group. All data is presented as mean values ±95% confidence intervals; (^*^) indicates significant differences between catch trial ratio groups.

For the consolidation process following motor adaptation, we found differing results for the considered performance measures suggesting a different sensibility to detect consolidation. Considering the velocity vector correlation, we found that the consolidation process can be positively influenced by catch trials. When subjects learned a second interfering task in-between, consolidation was least impaired for a catch trial ratio of 30%. This was demonstrated by a similar performance development during the consolidation period of corresponding control and test groups for 30% catch trial ratio (Figure 8A). For lower catch trial ratio (0, 10, 20%), however, consolidation was impaired when learning an interfering second task as indicated by significant differences in the consolidation between corresponding control and test groups. Similarly, for 40% catch trial ratio such significant difference in the consolidation between control and test group occurred. Therefore, learning with an optimal amount of catch trials seems to make the subsequent consolidation process more resistant to interference compared to learning without catch trials or learning with an immoderate amount of catch trials. For the perpendicular displacement, we also found an influence of catch trials on the consolidation process (Figure 8B). We detected differences in the degree of impairment of the consolidation process between the various catch trial ratios showing strongest resistance to interference for 30% catch trial ratio. However, for this measure, there was no complete resistance to the interference caused by force field B for any test group. Furthermore, we did not find consolidation when considering the learning index suggesting a disruption of the consolidation process when adapting to an interfering force field B, regardless of the induced amount of catch trials (Figure 8C).

To our best knowledge, Overduin et al. (2006) provided the only study which also considered both the velocity vector correlation and the learning index. They observed consolidation for subjects receiving 20% catch trials for both measures. Shadmehr and Brashers-Krug (1997) also used catch trials (approx. 17%) and found consolidation within the ABA-paradigm. However, they only computed velocity vector correlation coefficients. The fact that we detected a reduced interference on the consolidation process using this measure merely for a higher catch trial ratio of 30% might be explained by the higher complexity of our reaching task. In contrast to the mentioned studies, we did not support the subjects' arms. This results in more degrees of freedom within shoulder and elbow joints to be controlled and thus in an increased task complexity. Maybe, an increase of the catch trial ratio up to 30% further emphasized its positive effects on the consolidation process, compensated for our increased task complexity, and therewith facilitated consolidation. However, a further increase of catch trial ratio up to 40% seems to impair consolidation as it increases uncertainty about the task.

The missing detection of consolidation as measured by the perpendicular displacement and learning index might be due to a lower sensitivity of these measures compared to the velocity vector correlation. In this connection it is noteworthy that Overduin et al. (2006) also detected a trend toward a difference between their 20% catch trial control and test groups which, however, turned out not to be statistically significant. It remains the question, why there should be a differing sensitivity between the considered measures. The underlying computations offer a possible explanation because the measures are based on different types of information. The perpendicular displacement and the learning index depend on positional data. The velocity vector correlation, however, uses velocity data and therefore also considers temporal factors. Moreover, the velocity vector correlation is a similarity measure which compares fielded movements to baseline movements recorded under null field conditions, whereas the perpendicular displacement and the learning index are measures of difference.

**Figure 9:**
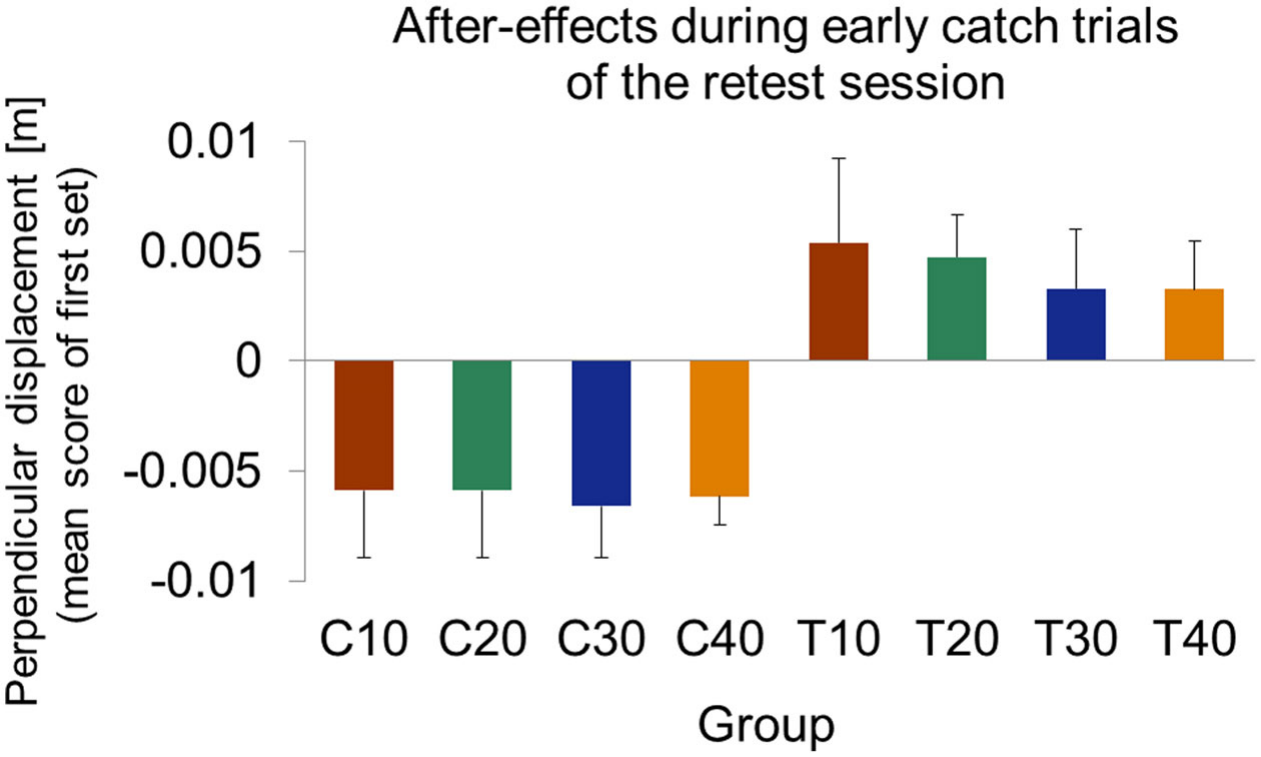
**Mean values of signed perpendicular displacement in catch trials of the first movement set at retest of force field A (day 3)**. All control groups show significant negative after-effect values indicating predictive force compensation appropriate to force field A. Test groups show significant positive after-effect values indicating predictive force compensation appropriate to force field B. All data is presented as mean ±95% confidence intervals.

Irrespective of methodological factors, the question remains, why test groups revealed differences in the reduction of interference that impaired the consolidation process. Former studies that investigated consolidation in ABA-paradigms discussed anterograde interference effects that might have avoided detection of consolidation (Brashers-Krug et al., 1996; Shadmehr and Brashers-Krug, 1997; Caithness et al., 2004). Anterograde interference describes the influence of the interfering force field B onto the recall of force field A. Therefore, anterograde interference might cover the consolidation of force field A (Robertson et al., 2004). We were able to detect anterograde interference for all test groups that received catch trials by considering the after-effects of learning force field B onto retest of force field A on day 3. Thereby, all considered test groups showed similar after-effects indicating anterograde interference of similar magnitude (Figure 9). Former studies using catch trials could not be sure about the relative magnitude of anterograde interference effects because only one catch trial group was given and for the 0% catch trial groups detection of after-effects was not possible. Certainly, we were not able to measure anterograde interference effects for our 0% catch trial test group T0 either. However, the similar after-effects of the catch trial test groups T10, T20, T30, and T40 allow extrapolation to the 0% catch trial group T0 and provide strong evidence for similar effects for all five test groups. Thus, anterograde interference effects do not depend on the induced catch trial ratio during adaptation. Therefore, anterograde interference cannot entirely explain the lacking consolidation. In our experimental design, test groups adapted to force field B on day 2 following the same protocol as on day 1. Thus, these groups received different amounts of catch trials leading to significant different attained end performances in force field B. This might have influenced the formation of an internal model appropriate to force field B. Therefore, the observed differences in the resistance to interference between catch trial ratios might be caused by the different types of interference induced via force field B. For the reason of comparability, our experimental protocol was designed similar to those of former studies presenting catch trials on day 2 (Shadmehr and Brashers-Krug, 1997; Overduin et al., 2006). To further enhance understanding of consolidation processes, future studies should keep the interfering task fixed.

As outlined above, subjects' attained end performance in force field A on day 1 decreased with increasing amount of catch trials. Nevertheless, all control subjects started with a similar level of initial performance at retest independent of the end performance on day 1. This finding emphasizes the importance to separate acquisition performance from retention performance (learning-performance distinction; Kantak and Winstein, 2012). Accordingly, challenging practice schedules with high variability induce difficulties for the learner which might impair acquisition performance but facilitate long-term retention and consolidation processes, referred to as the contextual interference effect (Schmidt and Lee, 2011). Research suggests, that variable practice schedules influence motor memory formation causing deeper cognitive processing and therefore stronger and more elaborate motor memory representations (Robertson et al., 2004; Kantak and Winstein, 2012). This positive effect is supposed to occur because variable practice induces more contrasting inter-trial comparisons than constant practice (Kantak and Winstein, 2012). In our design, catch trials produced variable practice schedules that led to impaired acquisition performance but a potentially stronger internal model representation of force field A enhancing long term retention. Altogether, there is strong evidence that the practice structure affects consolidation of motor memory (Robertson et al., 2004; Tanaka et al., 2009; Kantak et al., 2010; Kantak and Winstein, 2012). Moreover, in variable and constant practice, different neural substrates seem to be critical for consolidation (Tanaka et al., 2009; Kantak et al., 2010). Tanaka et al. (2009) further supposed that motor memories which are encoded in variable practice schedules are stored more quickly and become more rapidly stabilized and resistant compared to constant practice. These suggestions provide possible explanations for our findings on differing resistance to interference when learning with catch trials compared to constant force field practice. Yet, we suppose that for our specific task, not the presence of catch trials *per se* is important. Rather there seems to be an optimal amount of catch trial induced variability that facilitates the consolidation process.

### Comparison of motor adaptation and skill learning

At the outset of this article we argued that motor adaptation and skill learning need to be considered as two distinct features of motor learning (Huang and Krakauer, 2009; Krakauer and Mazzoni, 2011) and that the relationship between motor adaptation and skill learning is far from clear (Yarrow et al., 2009). In our view, comparing our results to the results from skill learning reveals some parallels.

Results in research on variability of practice showed that variable practice schedules in learning a single motor skill (McCracken and Stelmach, 1977) or multiple motor skills (Shea and Morgan, 1979) can lead to an impaired motor performance during the acquisition phase compared to constant practice schedules. These findings correspond to our results on motor adaptation. Hereby, groups with high variability induced by a high amount of catch trials showed a poorer performance at the end of the learning phase than groups with less or no catch trials. Taken together, in both cases, schedules with high variability that cause interferences lead to poorer performance at the end of the learning phase compared to groups that practiced under constant conditions.

As mentioned above, it is important to separate acquisition performance from retention performance (Kantak and Winstein, 2012). Again, this was shown when learning a single motor skill (Shea and Kohl, 1991) and multiple motor skills (Shea and Morgan, 1979). In our study, we observed a similar phenomenon, since groups practicing in intermittent practice schedules (high amount of catch trials) revealed impaired performance at the end of adaptation phase but the induced variability partly facilitated consolidation. However, the results of our study indicate that for motor adaptation there seems to exist an optimal amount of such variability. Nevertheless, these parallels have to be considered with caution and further research comparing both features of motor learning is required.

## Conclusion

In this paper we investigated the influence of catch trials on the overall motor adaptation process as well as on the consolidation process. We found that in motor adaptation, subjects show different ability to form an internal model depending on the amount of catch trials. These findings demonstrate a substantial influence of catch trials on the adaptation process. The consolidation process following motor adaptation further seems to be influenced by variable practice schedules suggesting an effect on the reduction of interference. For our specific task, a catch trial ratio of 30% was most beneficial indicating the existence of an optimal amount of catch trials. However, we cannot state a total absence of interference for any catch trial ratio. Moreover, the detection of consolidation seems also to be biased by the applied measure of performance. Therefore, further studies should account for the characteristics of used analytical methods. Comparing our results to results from motor skill learning (e.g., Schmidt and Lee, 2011) revealed similarities indicating that the processes of motor adaptation and skill learning possibly follow similar principles. However, similarities and differences between these two processes of motor learning should be focused in future studies to gain a more comprehensive understanding of human motor learning.

### Conflict of interest statement

The authors declare that the research was conducted in the absence of any commercial or financial relationships that could be construed as a potential conflict of interest.
